# Generalized Theorems for Nonlinear State Space Reconstruction

**DOI:** 10.1371/journal.pone.0018295

**Published:** 2011-03-31

**Authors:** Ethan R. Deyle, George Sugihara

**Affiliations:** Scripps Institution of Oceanography, University of California San Diego, La Jolla, California, United States of America; Governmental Technical Research Centre of Finland, Finland

## Abstract

Takens' theorem (1981) shows how lagged variables of a single time series can be used as proxy variables to reconstruct an attractor for an underlying dynamic process. State space reconstruction (SSR) from single time series has been a powerful approach for the analysis of the complex, non-linear systems that appear ubiquitous in the natural and human world. The main shortcoming of these methods is the phenomenological nature of attractor reconstructions. Moreover, applied studies show that these single time series reconstructions can often be improved *ad hoc* by including multiple dynamically coupled time series in the reconstructions, to provide a more mechanistic model. Here we provide three analytical proofs that add to the growing literature to generalize Takens' work and that demonstrate how multiple time series can be used in attractor reconstructions. These expanded results (Takens' theorem is a special case) apply to a wide variety of natural systems having parallel time series observations for variables believed to be related to the same dynamic manifold. The potential information leverage provided by multiple embeddings created from different combinations of variables (and their lags) can pave the way for new applied techniques to exploit the time-limited, but parallel observations of natural systems, such as coupled ecological systems, geophysical systems, and financial systems. This paper aims to justify and help open this potential growth area for SSR applications in the natural sciences.

## Introduction

A growing realization in many natural sciences is that simple idealized notions of linearly decomposable, fixed equilibrium systems often do not accord with reality. Rather, empirical measurements on ecosystems, metabolic systems, financial networks, and the like suggest a more complex, but potentially more information-rich paradigm at work [1]–[14]. Despite a long history of linear methods development in the engineering sciences, natural systems are generally not well described as sums of independent frequencies that can be sensibly decomposed, analyzed as non-interacting, and reassembled (e.g. Fourier or spectral analysis) in the style of traditional reductionism [15], [16]. Rather, quantitative measurements show many systems to be fundamentally non-equilibrium and unstable, in a manner more consistent with nonlinear (state dependent) dynamics occurring on a strange attractor manifold 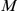, where relationships between state variables cannot be studied independently of the overall system state [17]–[27]. This emergent comprehensive view may help explain why many natural systems, such a those mentioned above, appear so difficult to understand and predict. Mirage correlations are commonplace in nonlinear systems where the manifold may contain trajectories that can temporarily exhibit positive correlations between variables for surprisingly long time periods (and in some regions of the state space) and can subsequently and rapidly exhibit negative correlations or no relationship in other time periods (and other regions of 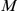). This transient property of apparent non-stationarity in correlations is one of the confounding phenomena faced by traditional linear models that require continual refitting and exhibit little or no predictive power.

In this paper, we present two general theorems that addresses the problem of characterizing the coupled dynamics of nonlinear systems using time series observations on a manifold 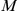. A special case of this theorem, attributed originally to Takens [12], provided the first sketch of a mathematical proof for reconstructing a diffeomorphic shadow manifold 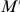 using lags of a single time series as coordinate axes. The basic idea, that was earlier demonstrated by Packard, Crutchfield, Farmer, and Shaw [28] and Crutchfield [2], is that under generic conditions, a shadow manifold 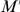 can be created using time-lagged observations of 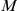 based on a single observation function (Cartesian coordinate variable) that is a smooth and smoothly invertible 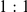 mapping with 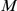. Subsequently, Sauer, Yorke, and Casdagli [29] provided a definitive proof and an explicit extension of Takens' theorem to fractal sets; their theorems are also more powerful than the original theorem, as they show embeddings are not just generic in the sense of being open and dense in the set of all mappings, but in fact almost every mapping in the sense of prevalence [30] is an embedding (see [30] for an in-depth explanation of the advantages of “prevalence” over “generic”). The theorem was also extended by Stark, Broomhead, Davies, and Huke [31], [32] and Stark [33] to include certain classes of stochastic systems. Practical methods for reconstruction have also been explored, particularly to address the presence of noise in real data (e.g. [29], [34]). Casdagli et al. [35] give a thorough treatment of such techniques based on transformations of univariate maps, showing how optimal noise reduction can be achieved. These very important prior results all focused on reconstruction from a single time series; however, as proven below, they can be extended to the more practically significant case where multiple observation functions are used to generate 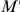.

Here we prove the more general case of multivariate embeddings (embeddings using multiple time series and lags thereof), and show how time series information can be leveraged if multiple time series and their lags are used to construct embeddings of 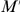. These theorems pave the way for more extensive use of state space reconstruction methods in practical applications where long time series may not be available, so that multiple diffeomorphic embeddings may be created in factorial fashion to more fully exploit the coupled non-redundant information that can be extracted from multiple time series (multiple observation functions of dynamics on a manifold) to create predictive shadow manifolds [36]. The use of multiple time series allows the possibility of noise reduction that exceeds the limitations of univariate reconstructions in the presence of noise [35].

The possibility of extending Takens' theorem to allow lags of multiple observation functions was mentioned in Remark 2.9 from [29], but was not explicitly proven. The remark was also restricted to mappings strictly formed from consecutive lags, which is not the only possibility that needs to be considered in the multivariate case. Given the potential importance of multivariate reconstructions, we believe a full proof is required—in particular, one that extends the generalization to non-consecutive lags. We show how Takens' theorem is a special case of our more general Theorem 2 (below) and by following the structure of Takens' original proof we clarify the logic and highlight the restrictions and special cases (non-generic cases) that can arise in its application to real world systems. We then give explicit proof of a stronger version of Remark 2.9 from Sauer et al. [29] that allows non-consecutive lags. This third theorem is stronger than the first two in the sense that it shows embeddings are prevalent and not just generic. For those less familiar, we begin with a brief overview of some basic terms and concepts used in our proofs.

### Some Basic Concepts of Embedding Theory

**Figure 1:**
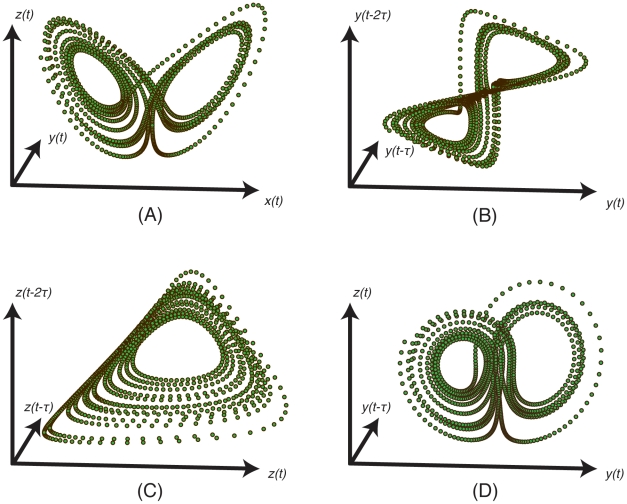
Lorenz attractor with three shadow manifolds. The Lorenz attractor [37] is shown with three shadow manifolds created from lag-coordinate transformations. The typical parameters were used: 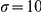, 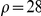, and 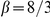, giving the three coupled equations as 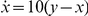, 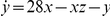, and 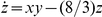. The solution was computed using a fourth order Runge-Kutta method with a time step of 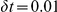, and the time lag used to create the shadow manifolds was 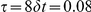. (A) The trajectory shown in the 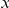, 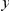, and 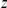 coordinates of the original system reveals a two-lobed manifold. (B) A univariate transformation using time lags of the 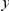-coordinate, 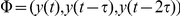, preserves this two-lobed structure (and other topological properties), verifying Takens' theorem. (C) A univariate transformation using time lags of the 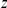-coordinate, 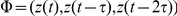, does not preserve the two-lobed structure. Local neighborhoods of the original attractor are, however, preserved. Thus, though this mapping violates a genericity assumption of the original theorem and is not an embedding, it is an immersion of the original manifold. (D) A multivariate transformation using both the 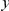
- and 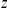-coordinates, 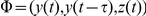, fulfills the assumptions of Theorems 2 and 7. As predicted, it also preserves the two-lobed structure of the Lorenz and is a valid embedding.

Consider the classic Lorenz attractor [37] shown in Figure 1(a), consisting of trajectories in three-dimensional space that together define a butterfly shaped surface or *manifold*. For simplicity, a manifold can be thought of as a generalized, *n*-dimensional surface embedded in some higher dimensional space, where the dimension of the manifold may be fractal (as is the case for the Lorenz attractor). More generally, an *embedding* is a multivariate transformation of a manifold that resolves all trajectories on the original manifold without crossings. That is, an embedding is globally 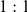 in that it resolves all *singularities* in trajectories that define the manifold (singularities are points on the manifold where trajectories cross so that future paths are not uniquely determined).

An *immersion* is a local embedding that may not preserve the global topology of a manifold. Rather an immersion preserves the topology of every local neighborhood of the original manifold, so that each point of the tangent space of the immersed manifold has the same dimensionality as the true manifold. Thus, an immersion is a mapping that is 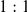 between any given “piece” of the true manifold and the immersed manifold. However, this condition does not guarantee that the global topology is preserved. This is illustrated in Figure 1(c), where two different pieces of the original manifold are mapped to the same piece of the immersed manifold, producing an immersion that is not an embedding. Immersions are nonetheless a useful conceptual stepping stone for constructing proofs about embeddings, since all embeddings are necessarily immersions.

The Lorenz attractor, Figure 1(a), provides an excellent example to illustrate both of these concepts. Consider two different multivariate functions that transform the original manifold, 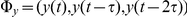 and 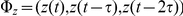 where 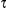 is a small time lag as in Takens' theorem. Both of these functions map points on the true manifold to points on a shadow manifold, shown in Figures 1(b) and 1(c). Examining these shadow manifolds, it is evident that both are immersions of the Lorenz attractor, because zooming in on a particular piece of either will reveal that the tangent spaces have the same dimensionality as the original. However, only Figure 1(b) is an embedding that successfully reproduces the two lobes of the butterfly. The reconstruction in Figure 1(c), based on lags of the 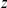-coordinate, fails to do so, because the two fixed points of the original attractor have the same 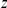-coordinate; they are mapped to the same point on the shadow manifold, so the two lobes are stacked on top of each other. This singularity is a consequence of a special, non-generic symmetry in the Lorenz system that violates an assumption of Takens' theorem. Figure 1(d) shows an embedding based on lags of both 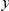
- and 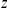-coordinates and is an example of the generalized mappings addressed in this paper.

## Results

### Two Theorems in the Style of Takens: The Generic Case

Let 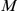 be a compact manifold of dimension 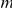. A dynamical system is a diffeophorism 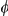 defining the trajectories or “flow” on 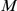 for discrete time or a vector field 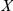 on 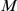 for continuous time. Takens [12] proved generically that given 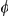 and 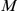, a smooth observation function 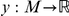 can be used to construct an embedding of 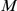 in 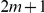 dimensions under the transformation 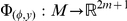 where 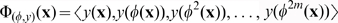. Here the components 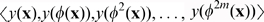 correspond to time-lagged observations of the dynamics on 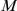 defined by 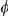. Notice that such mappings involve a single distinct observation function (i.e. a single time series), and represent a small subset in the larger set 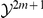
of all possible mappings 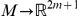 that could, for example, involve multiple time series and their lags.

Takens explicitly refers only to the unlagged 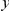 as an observation function, but in its most general sense an observation function is any 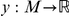. Thus, the functions 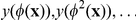, corresponding to the lags of the time series are technically observation functions as well. This bears mention, because in the more general case of mappings 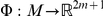, the observation functions making up the components of 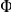 are not all derived from a single time series, but can be various lags of multiple time-series. To treat these cases, it is necessary to acknowledge that these are all observation functions, and we will refer to distinct time series as “unlagged” observation functions.

For a mapping 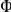 in the larger set 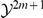 of all mappings 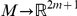, consider the case with 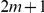 component functions 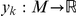 which are multiple unlagged observation functions of 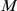 (i.e. multiple time series). Again, an observation function is any function 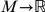 that assigns a real number to each point on the manifold 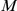. For a mapping 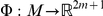, we can think of 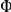 in terms of its 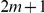 component functions, which correspond to the coordinates in 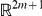. These component functions may all be lags of a single distinct observation function tracking a dynamical system, as in the case of Takens, or they may be multiple observation functions, as in the case of Whitney, or they may be lags of multiple observation functions, as in Theorems 2 and 7 below.

The question arises whether general multivariate mappings 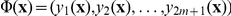 form legitimate embeddings. Here we present two theorems: one that demonstrates that maps created from 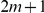 distinct observation functions are generically embeddings and another that shows that maps created from lags of multiple observation functions are also generically embeddings. Both of these theorems generalize Takens' theorem for which the component functions only involve a single observation function.

It follows from Whitney [38] that generically 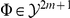 is an embedding. Note, however, that Whitney's work does not apply to the specific subsets of 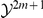 involving fixed lagged relationships as discussed by Takens for reconstructing attractor manifolds 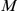 for dynamic systems. That is, Whitney's theorem is generic and does not address these specific subsets of 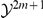 which have “measure zero” (e.g. in the sense of “shy” defined in [30]). To tackle this problem, we look to the proof of Takens and see that it can be readily generalized to the other subsets of 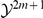, including the case of generic 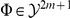.

Recall that, for a compact manifold, a mapping that is an immersion and injective is also necessarily an embedding. Thus, Takens' general approach was to first show that (i) immersions are dense in the set of mappings 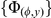, then that (ii) there is a dense set of 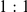 mappings within this set of immersions. Since the set of embeddings is open in the set of all possible mappings, Takens concludes that mappings in 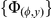 are generically embeddings. The critical word here is “generically,” meaning there can be exceptions (and as explained in [30], the set of such exceptions doesn't necessarily have zero measure).

To demonstrate both (i) and (ii), Takens argues that even when the property of interest (e.g. the 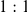 property) does not hold for some particular mapping, by making an arbitrarily small perturbation, it is possible to find a nearby mapping for which that property holds. The key to the theorem and also to adapting it to other sets of mappings is finding how to make these perturbations. The proof is most straightforward for the general case involving 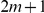 distinct observation functions (each a distinct time series) because it is possible to perturb the component functions of 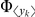 independently. Thus we begin with this proof to add clarity to the more powerful main theorem 2 involving lags of multiple observation functions.

#### Theorem 1

*Consider a compact,* 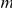 *-dimensional manifold* 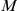 *and a set of* 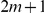 *observation functions* 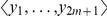 *, where* 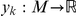 *smoothly; by “smooth” we mean at least* 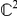 *. Then it is a generic property of all possible* 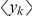 *that the mapping* 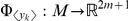 *defined as*

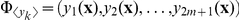

*is an embedding.*

#### Proof

Consider an arbitrary set of 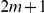 observation functions 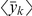 on 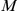. We define a corresponding mapping 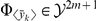 by letting each of these 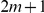 observation functions be one of the component functions of 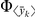. Now, recall that an immersion is a map with a derivative that is globally injective, i.e. 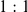. We denote the total derivative of a function 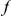 as 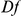. If the derivative is evaluated at a particular point 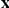 in the domain of 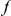, we will write 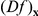, and if 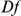 is a matrix, then we denote the derivative at a particular point and along a particular tangent vector 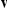 as 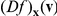.

For any point 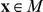, we can perturb the co-vectors 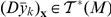 independently by perturbing individual 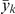. By making infinitesimal perturbations at points 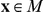 for which 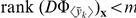, we can get a set of observables 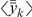 arbitrarily close to 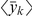 such that 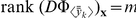 for all 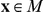
—i.e., 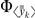 is an immersion. Since the set of immersions is open in the set of all mappings, there is a neighborhood 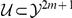 around this 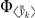 such that every 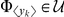 is an immersion.

Since immersions are local embeddings, we can find a 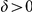 such that on the manifold, 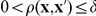 implies 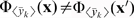. Here we depart from Takens' notation and let 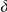 denote infinitesimal separations between two points on the manifold 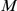 to avoid confusion with the later defined 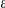 which is used to perturb the observable; 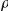 is any fixed metric on 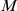. In fact for this fixed 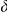, there is a subset 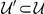 such that for any 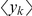 in 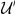, the associated map 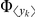 is an immersion, and 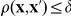 implies that 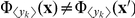.

Next, we show that we can find a globally 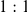 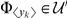 arbitrarily close to 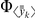 . To do this, we construct a finite collection of subsets 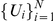 such that the 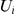 are open subsets of 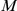, the collection covers 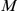, and 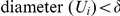 for every 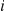. Then, we take a partition of unity 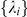 corresponding to these 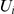, so that we can vary the value of any 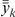 by an infinitesimal amount 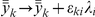 without altering the value of 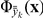 for 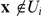.

We now consider the mapping 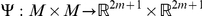 defined as 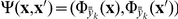. We define the set 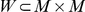 as 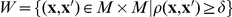, so that (by our choice of 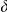), the mapping 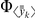 is necessarily injective on the complement of 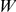 in 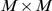. Furthermore, note that the intersection of 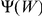 with the diagonal of 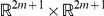 gives the set of points 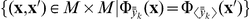, and therefore 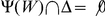 is equivalent to 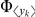 injective. Our task, then, is to perturb the manifold 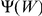 using the 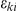 and 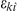 so that it does not intersect the diagonal manifold 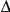.

At each 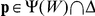 we know that 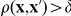, so 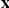 and 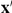 cannot belong to the same 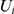. Consequently, varying an 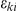 or 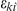 only alters the value of 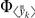 at either 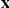 or 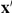 (respectively). In the tangent space 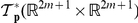, then, the direction of the 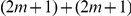 infinitesimal changes given by the 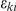 and 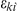 are all linearly independent (indeed orthogonal) and as such span 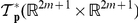. Since the tangent spaces of 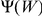 and 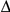 are at most 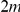 and 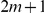 dimensional, respectively, we can construct a vector from a linear combination of 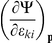 and 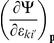 that lies outside of both 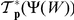 and 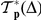. Therefore, an infinitesimal perturbation corresponding to this linear combination will move the sub-manifolds 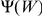 and 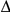 away from each other at the point 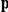 without creating a new intersection at another point. By keeping the size of these perturbations sufficiently small, we ensure that we stay confined to 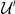, so that 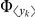 is still an immersion. This is a more transparent statement of the transversality argument used in the Takens proof (1981).

Thus, we have shown that for any arbitrary set of 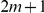 observables 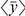, we can find a set of observables 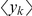 arbitrarily close to 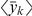 such that 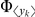 is an embedding—i.e., there is a dense set of observables 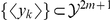 such that 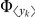 is an embedding. The set of embeddings is open in the set of all mappings, so this set is dense and open, meaning that the embedding property is generic over all mappings.

When mappings are confined to fixed lag relationships, Takens showed it is valid to independently perturb each component of 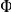 at a given point of the domain by perturbing the unlagged observation function, 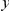, in the other parts of the domain corresponding to neighborhoods of the lagged states 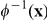, 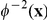, etc. This ensures that the perturbations to 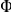 maintain the structure of the lag relationships and that we have not inadvertently left the subset of interest. As we now show, this allows the above result to be easily extended to families of maps having component functions that are the lags of multiple observation functions. This is the relevant case for many practical examples where lags of multiple time series (multiple variables or observation functions) are required to achieve a mechanistic reconstruction of 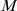 (e.g. [20]). It also allows information on 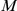 to be leveraged when the time series are short, as is the case in many physical and biological problems [22], [36].

Before starting the proof, however, we must clarify exactly what the “subsets of interest” are. We define these sets as follows. First, we say 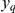 is a lag of the observable 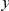 if we can write 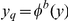 for positive 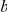. We consider the lags in the positive time direction only to simplify notation in the proof, noting that the results apply equally to negative lags. Let 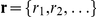 be the subset of 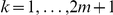 for which 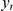, 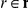 is an unlagged observable, i.e. 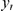 is not a lag of another 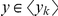. We begin with the “unlagged” observation functions, 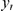, or observation functions that are not a lag of another observable in 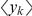. Now define a set 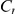 for each 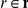 that contains 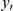 and any other observation function in 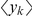 which is a lag of it. That is, 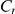 is the set of 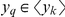 that are lags of 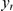 given as 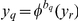, where the lags 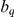 are distinct for fixed 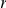. This choice of 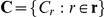 and 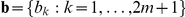 determine a subset 
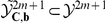
 containing all choices of 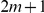 observables 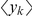 which obey the correct lag relationships under a dynamical system 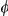. Note that each element of 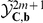 can be identified by the dynamical system and the 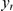. We denote such an element, then, as 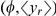.

#### Theorem 2

*Consider a diffeomorphism* 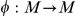 *on some compact manifold* 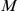 *of dimension* 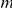 *, along with* 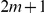 *observation functions* 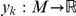 *, smoothly; by “smooth” we mean at least* 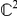 *. Restrict the* 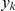 *to have the lag relationships corresponding to a collection of sets* 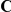 *and lags* 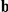 *under the dynamical system* 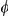 *, and impose the following generic* [12], [39] *properties on* 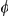:

1. *The set* 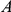 *of periodic points with period* 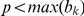 *has finitely many points,*
2. *The eigenvalues of* 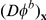 *at each* 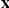 *in a compact neighborhood* 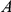 *are distinct and not equal to 1.*

*Then, for generic* 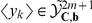 *, the mapping described by*

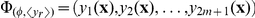

*is an embedding.*

#### Proof

The proof of this theorem closely follows the logic of the previous proof and the original argument of Takens [12]. As noted above, any perturbations to 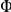 via its component functions 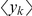 must remain within 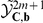 (the set of observables having the desired lag relationships under 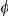 prescribed by the 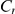 and the 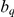). Here we must also deal with points of 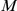 that are fixed points or periodic under the dynamical system 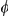, i.e. the points for which there exists a 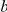 such that 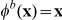 (including the fixed point case, 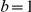). The above proof shows that the mapping 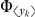 is generically an immersion because the co-vectors 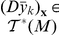 can be independently perturbed. This is also true for non-periodic points where there are fixed lag relationships between some observables, as we can perturb 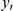 in the neighborhood of 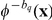 and thus perturb 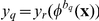 without affecting 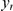 in the neighborhood of 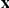.

Note that periodic points 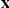 can exist such that the period 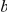 or some integer multiple of it, 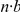, is the fixed time lag between two observables 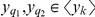 belonging to the same 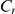. Let 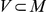 be a compact neighborhood of all such points. For 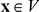, the vectors 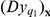 and 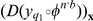 cannot necessarily be perturbed independently. Nonetheless, while 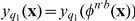 for such a point, it is not generally true that 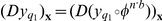. By assumption, for each 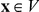, the eigenvalues of the 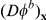 are distinct and not equal to 1. Thus, by the chain rule, it is clear that 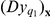 and 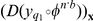 are linearly independent. As noted above, all the other 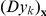 can be perturbed independently, so we can find a set of observables 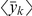 arbitrarily near 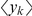 in 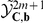 for which 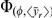 is an immersion on 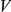. Note that because the set of immersions is open, there is an open neighborhood in 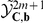 around this 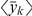 for which every set of observables in that neighborhood gives an immersion.

We must also satisfy 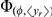 injective. The proof above relied on the ability to independently perturb the manifold 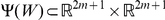 at any point 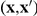 by an infinitesimal amount in any coordinate direction. For a periodic point on 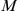 with perioid 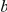 and two observables related as 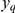 and 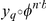, it is impossible to independently perturb 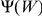 locally in the coordinate 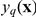 or 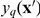, as you also perturb 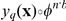 or 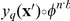. By assumption, the set 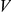 has a finite number of elements. For such a generic 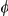 and any set 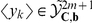, any neighborhood of the 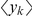 will contain a set of observables 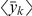 for which the unlagged observation functions 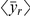 take distinct values at each point in 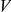.

We first perturb the 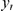 to find an open neighborhood of observables which give immersions when restricted to the set 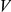. We then further perturb the observables to find within this neighborhood a set of observables 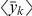 for which 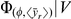 is also injective and therefore an embedding (on 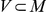). Since embeddings are dense in the space of all mappings, there is a neighborhood 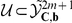 such that for all 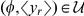, the map 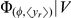 is an embedding.

We now show that we can find a 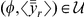 such that 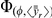 is an embedding on *all* of 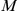. We first note that at points 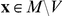, the vectors 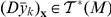 can be perturbed independently, so we can find 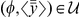 for which 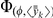 is an immersion. Because an immersion is a local embedding, there is a 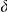 such that for 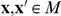, 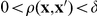 implies that 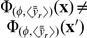. Since the set of immersions is open in the set of possible mappings, there is a neighborhood 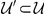 such that for any 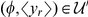, the corresponding mapping 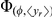 is an immersion. Thus, for the same 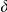 as above, 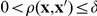 implies 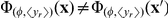.

Now we need to show that there is a 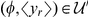 such that 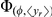 is also injective on 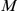. As noted in the first proof, this is equivalent to 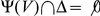 for the mapping 
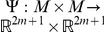
 defined as 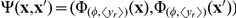. If 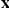 and 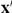 are both in 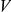 or 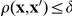, we already know that 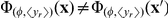. Thus we restrict ourselves to the set 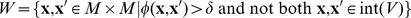.

To perturb the manifold 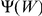 away from 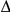 at points of intersection, 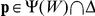, we must be able to find variations for which the tangent vector 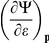 is linearly independent from the 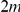 tangent vectors 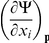 and 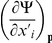 and lies outside of 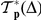. In the first proof, it was obvious that each component of 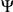 could be perturbed independently. Now we must be more careful. We do this by first creating a collection of 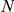 open subsets of 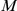, 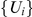, with the following properties:

1. The 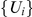 cover the closure of 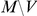.
2. For each 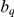 and 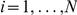, the diameter of 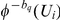 is less than 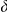.
3. For all choices of 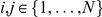, the set 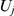 intersects with 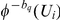 for at most one 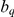.
4. For 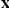 and 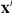 such that 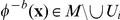 for some 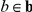, 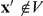, and 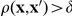, no two of 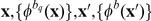 belong to the same 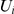.

Take a partition of unity 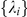 corresponding to this 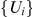. Because of the way we constructed the 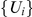, we can vary the value of each 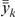 at any point 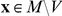 by an infinitesimal amount without altering the value of the other 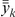 in the neighborhood of 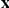. We make this explicit as follows. To perturb the 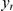, we take 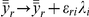 for 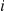 corresponding to 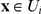. To perturb the other 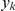 (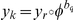 for some 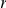), we perturb 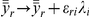 for 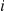 corresponding to 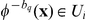 . Consider the 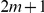 perturbations, 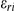, which are independent shifts at 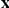 in distinct 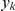. In 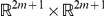, we note that each corresponding tangent vector 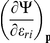 lies outside of 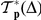. Note the 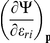 together with any basis of 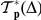 form a linearly independent set of vectors. Since the dimension of 
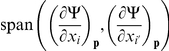
 is at most 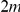, there must be a linear combination of the 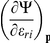 that lies outside of both 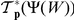 and 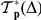, which can be used to perturb 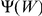 away from 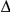. By keeping variations in the 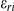 sufficiently small, we can find a set of 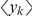 such that 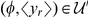 and 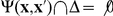 (where 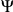 now corresponds to the 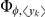 map). This pair gives a mapping 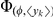 that is both an immersion and injective, and thus is an embedding. Because 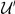 was an arbitrarily small neighborhood of any point in 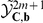, this means embeddings are dense in 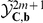, and the set of embeddings is open in the set of mappings. Thus, the map 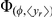 given by 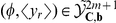 is generically an embedding.

Just as Takens extends the original result for discrete time to dynamical systems in continuous time, we can extend our result as follows:

#### Corollary 3

*Consider a smooth vector field* 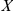 *on some compact manifold* 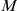 *along with* 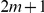 *observables* 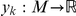 *, smoothly; by “smooth” we mean at least* 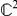 *. Define* 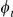 *as the flow on* 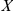 *. Suppose we restrict the* 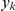 *to have the lag relationships corresponding to a collection of sets* 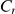 *and lags* 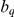 *under the discrete dynamical system* 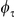 *, where* 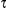 *is a constant. We impose the following generic properties on* 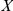 *:*

1. *For points* 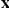 *such that* 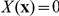 *, the eigenvalues of* 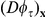 *are distinct and not equal to 1.*
2. *No periodic integral curve of* 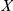 *has integer period* 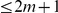 *.*

*Then, for generic* 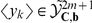 *, the mapping described by*

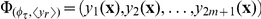

*is an embedding.*

#### Proof

In this case, 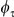 is a discrete time dynamical system on 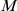 satisfying the conditions imposed in the theorem above, and this corollary follows directly.

### A Theorem in the Style of Sauer et al.: The Prevalent Case

We now give an explicit proof of Remark 2.9 from [29] using the framework constructed in their original paper, but we extend the language to cover reconstructions using non-consecutive lags (from multiple time series). The proof uses Lemma 4.1, 4.6, and 4.11 from [29] to show that 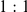 mappings and immersions are prevalent in the space 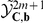, just as Sauer et al. use Lemma 4.6 to prove Theorem 3.3, and Lemmas 4.1 and 4.11 to prove Theorem 3.5. These lemmas are now stated (for the proofs, see their original paper).

#### Lemma 4

*(Originally part 2 of 4.1) Let* 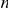 *and* 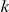 *be positive integers,* 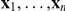 *distinct points in* 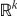 *,* 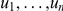 *in* 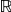 *, and* 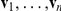 *in* 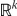 *. Then there exists a polynomial* 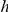 *in* 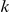 *variables of degree at most* 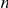 *such that for* 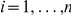 *,* 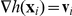 *.*

#### Lemma 5

*(Originally 4.6) Let* 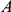 *be a compact subset of* 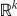 *. Let* 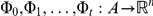 *be Lipschitz maps. For each integer* 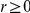 *, let* 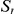 *be the set of pairs* 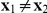 *in* 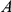 *for which the* 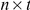 *matrix*

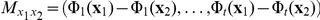

*has rank* 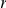 *, and let* 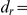 *lower boxdim* 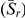 *. Define* 
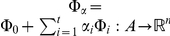
 *. If* 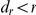 *for all integers* 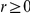 *, then for* 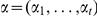 *outside a measure zero subset of* 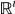 *, the map* 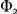 *is* 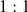 *.*

#### Lemma 6

*(Originally 4.11) Let* 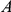 *be a compact subset of a smooth manifold embedding in* 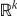 *. Let* 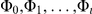 *be a set of smooth maps from an open neighborhood* 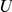 *of* 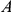 *to* 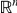 *. For each positive integer* 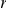 *, let* 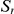 *be the subset of the unit tangent bundle* 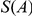 *such that the* 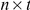 *matrix*

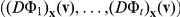

*has rank* 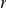 *, and let* 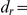 *lower boxdim* 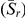 *. Define* 
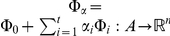
 *. If* 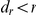 *for all integers* 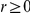 *, then for almost every* 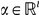 *, the map* 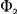 *is an immersion on* 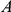 *.*

To apply these lemmas, it is necessary to restrict the dimension of the sets of periodic orbits—that is, the sets 
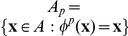
 for 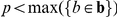. For the case of consecutive lags, Sauer et al. state sufficient conditions to be boxdim 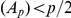. A sufficient condition for non-consecutive lags is a bit more complicated. Define the constants 
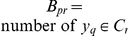
 such that 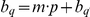 for at least one 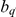 and 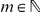. Also, define 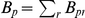. A sufficient condition on the 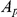 is 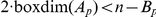.

#### Theorem 7

*Let* 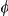 *be a diffeomorphism on an open subset* 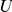 *of* 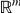 *, and let* 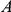 *be a compact subset of* 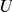 *,* 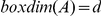 *. Let* 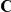 *be a collection of sets and* 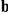 *a set of lag relationships as above, such that* 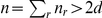 *. Assume that for every positive integer* 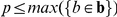 *, the set* 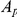 *of periodic points of period* 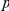 *satisfies* 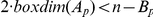 *, and that for each point of* 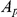 *, the Jacobian* 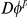 *has distinct eigenvalues. Then, for almost every set of* 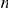 *observation functions* 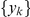 *satisfying the given lag relationships, the map*

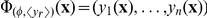

*is an embedding on* 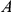 *.*

#### Proof

Without loss of generality, assume we have ordered the components of 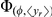 with 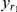 and all its lags first, then 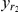 and its lags, etc. That is,
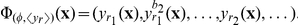
To show prevalence, we find a suitable probe space (see [29]). The infinite dimensional space for the univariate theorem is the observation functions 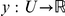, smoothly. For maps constructed from multiple lags, this becomes the sets of 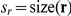 unlagged observation functions. Sauer et al. take the probe space for the univariate theorem to be any set 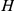 of polynomials in 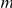 variables which include all such polynomials up to degree 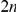. It is now necessary to have a set of polynomials for each of the 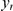. Thus, we take the probe space for this theorem to be the Cartesian product of 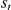 copies of 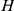.

Let 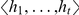 be a basis for 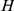. We want to show that for almost all choices of 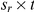 coefficients 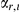, the map 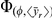 defined by the observation functions 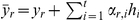 is an embedding. We first demonstrate that almost every 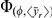 is 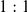, proceeding as in the proof of Theorem 4.3 in [29].

To sensibly apply Lemma 5, we adopt the following convention: think of 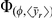 as a perturbation of 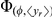, which is the summed effect of perturbations on each 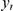 separately. For each pair 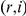, 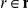 and 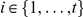, there is a map 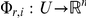 which is 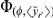 for 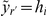 if 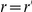 and 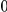 otherwise. The components of 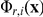 are either 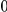 or of the form 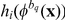. Consequently, 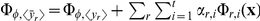, which matches the structure Lemma 5.

We now check that the rank of the matrix 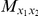 satisfies the conditions of Lemma 5 for each pair of distinct 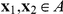. Note that to avoid confusion with the previous section of this paper and Takens' original work, we continue to use row vectors to describe the transformations 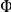. However, Sauer et al. [29] prefer column vectors, so it is necessary to use of transposes in several instances. Thus, we have
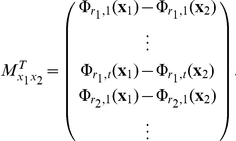
Note that 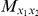 is a block diagonal matrix, and so it has rank equal to the sum of the rank of the blocks. Each of the 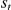 blocks can be rewritten as the product of two matrices, 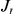 and 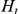, where the entries of 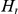 are values of a single polynomial 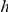 and the entries of 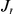 are each one of 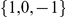. Note, there are multiple possible choices for 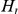 and 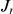 that give the same 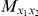.

*Case 1*: First consider 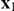 and 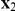 that do not both lie in a periodic orbit of integer period less than 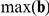. We specify 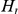 so that the first 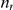 rows, where 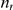 is the size of the set 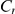, correspond to the 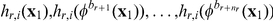, and the next 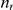 correspond to the 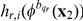. 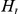 is onto, so the rank of 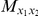 is just the sum of the ranks of the 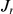. For this case, 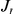 contains a copy of 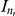, and thus will have rank 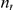. The entire matrix 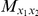 will thus have rank 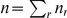, which satisfies the conditions of Lemma 5.

*Case 2*: Now consider 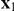 and 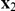 in separate periodic orbits with periods 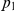 and 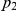 such that 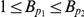 and 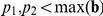. 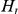 will have 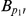 fewer rows corresponding to the 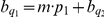 for some 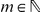 (there will also be a reduction in the number of rows associated with 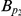). In this case, 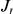 will still contain the column space of 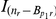 and thus 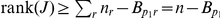. Again the 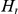 are onto, and so the rank of 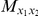 is the rank of 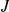.

The dimension of the set 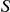 of all pairs 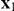 and 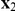 is 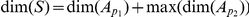. By the conditions placed on the size of the 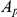, we can conclude that 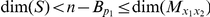, and thus that Lemma 5 applies to this case as well.

*Case 3*: Finally we consider 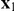 and 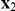 in the same 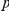-periodic orbit, 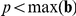. Now the matrix 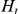 becomes more complicated, since some of the 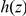 pertaining to 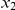 may be equal to 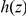 pertaining to 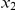. Consequently, the 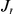 are no longer guaranteed to contain the column space of the identity. Each 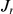 does contain the column space of an 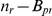 dimensional matrix with 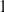 along the upper diagonal and a single 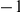 off the diagonal in each column. Using elementary operations, it is possible to make the first 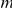 columns of 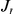 upper diagonal for some integer 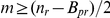. Thus, the rank of each 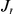 is at least 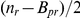 and the entire matrix has 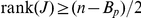.

The dimension of the set 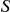 of all such 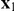 and 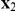 is just 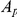. By the imposed conditions, 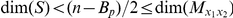, and Lemma 5 applies.

Now we want show that almost every 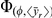 is an immersion. We check that the matrix
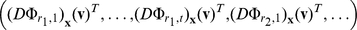
has full rank and thus satisfies the conditions of Lemma 6 for each 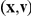 in the tangent bundle 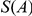. Note that this is a block diagonal matrix with 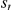 blocks, so it is sufficient to show that the columns of the 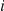
th block span the subspace 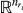 for 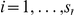. We consider two cases.

*Case 1*: Consider first the subset 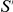 of 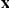 that are not periodic with period 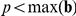. The entries of each block are of the form 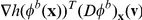. Since 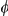 is a diffeomorphism and 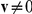, we know that 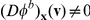. Furthermore, the 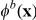 are distinct points. Examining Lemma 4, it is clear that the columns span 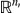. The dimension of 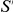 is at most 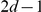, so we may apply Lemma 6.

*Case 2*: Now consider the subset 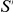 of 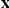 that are periodic with period 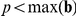. By the conditions of the theorem, 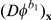 has distinct eigenvalues from 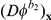. Therefore, 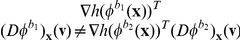. Furthermore, the relationship depends on 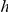, and again referencing Lemma 4, it is clear that the columns span 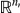. The dimension of 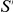 is certainly less than 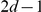, so we can safely apply Lemma 6.

Theorem 7 can be extended to continuous dynamical systems (smooth vector fields on a manifold) by letting the flow 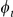 of 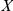 be 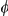 in the statement of the theorem.

## Discussion

Theorem 1 and the more general result presented in Theorem 2 (and its corollary) were given proofs intended to follow those presented by Takens. The original “transversality” argument, however, has been replaced with what we reckon is a simpler and more direct argument. These clarify how perturbations to the observation functions can be constructed and highlight why 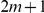 dimensions are necessary to have mappings that are generically embeddings. Theorem 7 is similar to Theorem 2, but takes advantage of the more powerful framework, built around the notion of prevalence, established by Sauer et al. [29]. It also provides more specific conditions on the periodic orbits than Theorem 2 and thus can be applied to certain non-generic situations that Takens' original framework would exclude. Namely, the set of periodic points need not be finite (as required in Takens' original theorem and Theorem 2), so long as the dimensionality does not exceed the bounds stated in Theorem 7. Theorem 7 is an extension of Remark 2.9 in [29], which we explicitly proved by determining a sufficient restriction for the periodic orbits when the lags composing 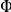 aren't necessarily consecutive.

This work also develops a language to describe a wider family of cases for reconstructing state space manifolds from multiple observational time series to encourage wider applicability of SSR in the natural sciences. For example, these results can be extended to another special case of interest for reconstructions using time derivatives [40], when multiple observation functions are available. The argument for this case is analogous to that used by Takens [12] for the case when all the derivatives are from a single observation function. Furthermore, these theorems validate heuristic work using spatial lag reconstructions and mixed spatial and temporal lag reconstructions to study spatially coupled dynamics [41].

More importantly, in terms of future applications, Theorems 2 and 7 set the stage for practical reconstruction of state space manifolds from multiple observation functions. This is significant in answering objections to single variable state space reconstruction (SSR) concerning the excessive phenomenology of lagged-coordinate embeddings [26]. These two theorems provide proof of principle for modeling attempts of nonlinear dynamics in the natural sciences involving multiple time series (e.g. [20]), and lays bare the rather non-restrictive assumptions required in such applications for building mechanistic models from multiple time series variables. Moreover, it gives support to the notion of using multiple embeddings as a potentially efficient way of extracting information from time series data of limited length, but where there are potentially many simultaneous observations of dynamics on the same attractor manifold. By reducing correlations in noise between the reconstructed coordinates, these techniques should allow reconstructions to exceed the limitations placed on univariate methods [35], as heuristic examples have already suggested [20]. The potential information leverage provided by multiple embeddings possible from novel combinations of variables (and their lags) can pave the way for a plethora of new applied techniques to exploit the time-limited, but parallel observations of nature [36]. This paper is intended to complement the existing literature on SSR and help promote this potential growth area in the natural sciences.
